# Approaching microwave photon sensitivity with Al Josephson junctions

**DOI:** 10.3762/bjnano.13.50

**Published:** 2022-07-04

**Authors:** Andrey L Pankratov, Anna V Gordeeva, Leonid S Revin, Dmitry A Ladeynov, Anton A Yablokov, Leonid S Kuzmin

**Affiliations:** 1 Nizhny Novgorod State Technical University n.a. R.E. Alekseev, GSP-41, Nizhny Novgorod, 603950, Russia; 2 Institute for Physics of Microstructures of RAS, GSP-105, Nizhny Novgorod, 603950, Russia; 3 Lobachevsky State University of Nizhny Novgorod, 603950, Nizhny Novgorod, Russia; 4 Chalmers University of Technology, 41296, Gothenburg, Sweden

**Keywords:** Josephson junction, microwave photons, single photon counter, thermal activation

## Abstract

Here, we experimentally test the applicability of an aluminium Josephson junction of a few micrometers size as a single photon counter in the microwave frequency range. We have measured the switching from the superconducting to the resistive state through the absorption of 10 GHz photons. The dependence of the switching probability on the signal power suggests that the switching is initiated by the simultaneous absorption of three and more photons, with a dark count time above 0.01 s.

## Introduction

The development of a single photon counter (SPC) for microwave frequencies of tens of gigahertz has been required for several applications at least for the last two decades. The difficulty of this development is in the small energy scale: The energy of a photon of 10 GHz is just 7 yoctojoule (7 × 10^−24^ J). To realize the detection, the photon must trigger a process whose energy is of the order of this value (the difference between initial and excited states). There are not many examples in solid-state physics with such energy scales. Another difficulty is that a spontaneous change of the state must be significantly less probable so that the detector could be in a waiting mode for a significant amount of time.

Superconductor–insulator–superconductor (SIS) junctions have not been seriously considered previously for the role of detectors of single photons in the microwave range, despite sporadic works showing such a possibility [1–7]. Recently, the interest in microwave SPCs has been increased [8–9] due to new experiments of dark matter search [10–12] and the corresponding program initiated by INFN in Italy [13–22].

Our experiments show that typical aluminium Josephson junctions (JJs) can indeed have a few-photon sensitivity in the microwave frequency range, and a photon counter can be made on their basis. We use the metastable quasi-equilibrium state of a Josephson junction, which, at low temperatures, is stable enough for thermal fluctuations and quantum tunneling, but can be easily destroyed by absorption of a single photon. We demonstrate few-photon sensitivity of our samples in a single-shot regime and outline the junction parameter range where approaching single photon sensitivity is possible.

## Results and Discussion

**Figure 1:**
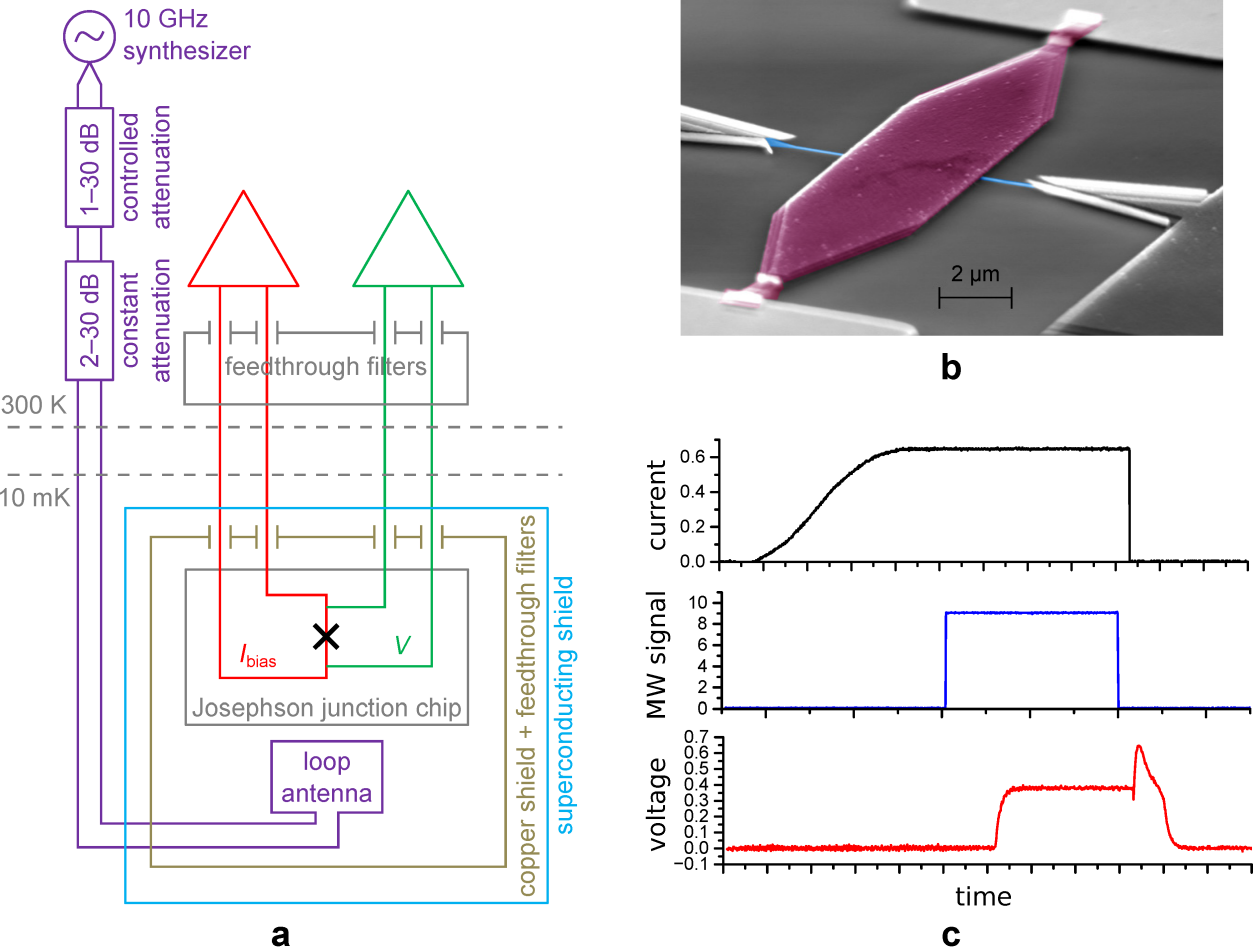
(a) Scheme of the measurement electronics with thermal anchoring and various filtering stages. (b) SEM image of the SIS junction. The top electrode is highlighted in magenta color, the bottom electrode (blue color) has the same shape as the top one in the area of the tunnel barrier. (c) Time diagram of the channels: current through the JJ, initial pulse modulation of the microwave signal (assuming front smoothing due to twisted pairs) and voltage across the JJ.

In this section, we describe our experimental setup, as well as the measurement results and comparison with theory. To study the dynamics of a SIS tunnel junction, we have thermally anchored the sample to the mixing chamber of a He3/He4 dilution refrigerator Triton 200 from Oxford Instruments. A block diagram of the experimental setup, including filtering and room-temperature electronics, is shown in Figure 1a. The sample (Figure 1b) was mounted in an RF-tight box with a superconducting shielding on the coldest plate. The dc bias wires were filtered with feedthrough capacitors at room temperature and RC filters at the 10 mK cryostat plate, minimizing the effect of unwanted low-frequency noise.

For an experiment with microwave radiation, we used phosphor bronze twisted pairs with an attenuation of −15 dB/m at 10 GHz to provide the radiation to the sample in the cryostat. The twisted pair ended with a loop antenna near the JJ, see [7,23] for the setup description. As any in-stock microwave synthesizer would produce a far too powerful signal, in our setup, we used constant attenuators from 2 dB to 30 dB and a voltage-controlled room-temperature attenuator, preliminarily calibrated with a spectrum analyzer. We altered the power of the signal from high power, at which the photon-assisted tunneling steps are well pronounced in the *I*/*V* curve [23], to low power, whose presence can be observed only in the switching distributions and in the shorter lifetime of the superconducting state.

The used experimental setup is the same as in [7], except for the measured sample. In [7], the critical current of the sample was very low, and the phase diffusion regime was noticeably pronounced. The sample considered here has a much higher critical current, and the phase diffusion does not appear. As a result, the theoretical estimates based on the BCS theory for critical currents and Kramers’ theory for escape times are well applicable. Furthermore, the analysis of the experiment is done with a more general theory, which considers the absorption probabilities of different numbers of photons.

The time traces of setting a current and an external microwave signal to measure the switching probability as a function of power are shown in Figure 1c. First, the current through the junction is increased up to the required value by a sin^2^ law [24] to realize a quasi-adiabatic ramping, then the microwave signal is turned on for a fixed time slot, assuming significant smoothing of pulse fronts due to transfer via twisted pairs. Due to strong attenuation of the harmonic signal, the microwave pulse represents a sequence of single photons, pairs, triples, and so on, which obey a Poisson distribution [25–26]. After turning off the signal, the state of the JJ is checked. Depending on whether the JJ is in the resistive or superconducting state, unity or zero is added to the switching probability, respectively.

**Figure 2:**
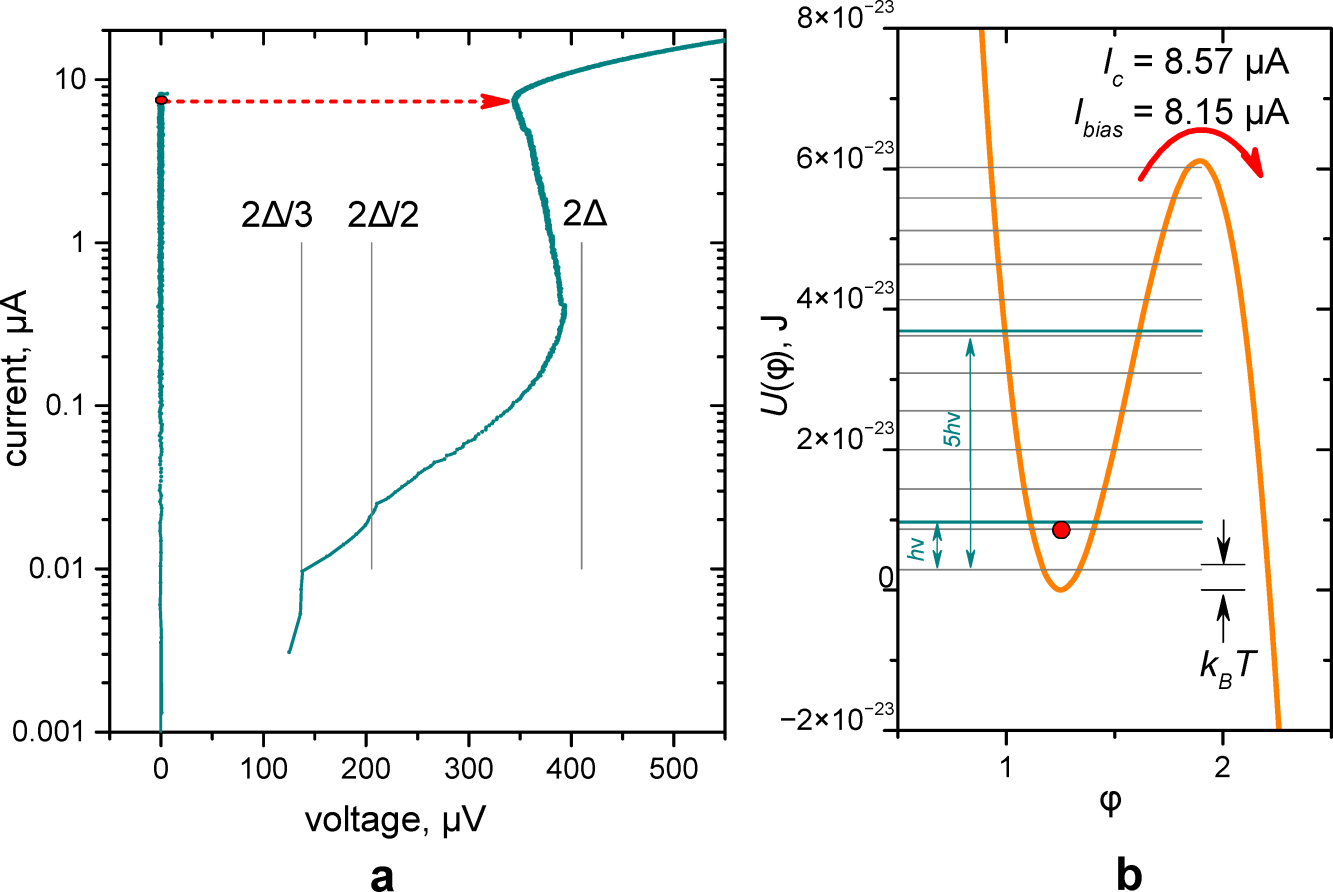
(a) The current–voltage characteristics of the Josephson junction with *I*_c_ = 8.6 μA at 50 mK. The red point indicates the state of JJ in a “waiting” mode, the arrow shows a jump to the resistive state after absorption of photons. (b) The potential profile at the bias current of 8.15 μA. The energies of one and five photons are shown by lines relative to the minimum energy level. Under these conditions, the JJ switches with a probability of 1 when five photons are absorbed simultaneously (*q*[5] = 1), and with a probability of 0.13 when four photons are absorbed (*q*[4] = 0.13). These probabilities are obtained from fitting experimental data, see Figure 5 below. The scale of the effective thermal fluctuation energy is given by black arrows for *T* = 265 mK (see the main text).

We begin our consideration of the Josephson junction as a photon counter with its current–voltage characteristic (see Figure 2a) and the determination of the critical current. All further analysis of experimental results and understanding of the energy relations of the JJ in comparison with the energy of photons (see Figure 2b) depends on the accuracy of determining the critical current. An area of 60 μm^2^ and a critical current *I*_c_ ≈ 8.6 μA have been measured for the Al JJ, see the SEM image of the sample in Figure 1b. Due to rather low-noise measuring environment, used before for terahertz receiver applications [27–28], in Figure 2a one can see a typical current–voltage characteristics (IVC) with the critical current close to the theoretical value [29]. Besides, a subgap structure is visible at the inverse branch of the IVC. Such a structure with peculiarities in the differential resistance at voltages 2Δ/*n* was calculated theoretically for normal metal links between two superconductors as multiple Andreev reflections [30] and observed in experiment both for superconductor–normal metal–superconductor and SIS junctions [31].

**Figure 3:**
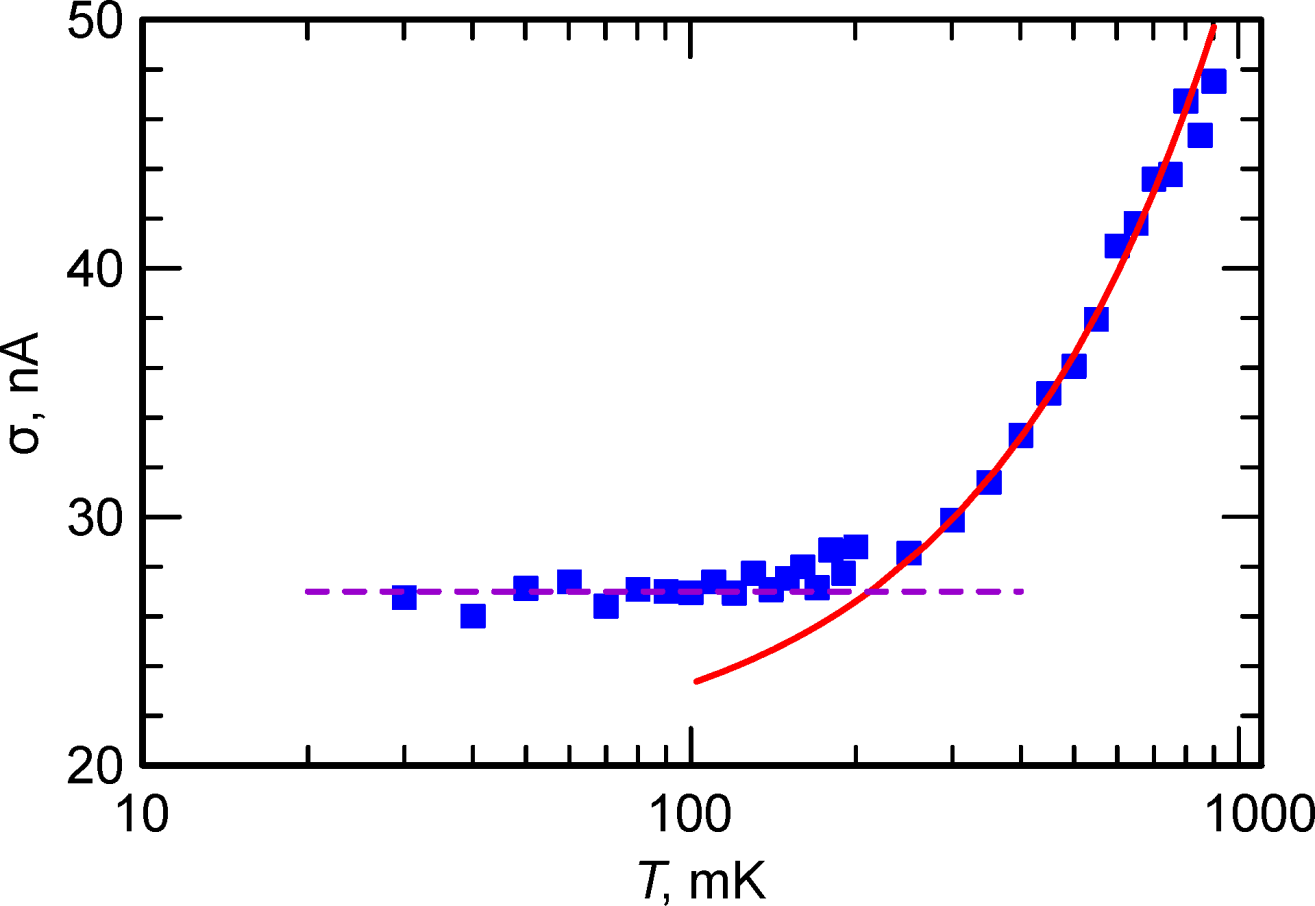
Width of the switching current distribution of the Josephson junction. One can see a standard behavior when the distribution width grows monotonically with increase of the temperature. Here, the violet dashed line shows the quantum regime and the red solid curve shows the thermal activation regime.

In contrast to smaller junctions [7], where the phase-diffusion regime is possible [32–37], the analyzed junction demonstrates a typical behavior [4,38], that is, a monotonic increase in the switching current distribution width with the rise of the temperature, see Figure 3. For the switching current measurements, the bias current of the junction was ramped up at a constant rate. The voltage was measured using a low-noise room-temperature differential amplifier AD745 and was fed to a high-speed NI ADC card. The switching current histograms were collected in the temperature range between 1 K and 30 mK. The dependence of their width on the temperature is shown in Figure 3. It is interesting to note the crossover temperature to the quantum regime of about 250 mK, which is somewhat lower than in [38] for junctions with larger critical currents.

The switching current to the resistive state depends on the sweep rate. Therefore, its value is underestimated in dc measurements. The upper limit is given by the BCS expression 1.75*kT*_c_/(*eR*_N_) [29], which depends on the critical temperature of the electrodes and the normal resistance of the tunnel barrier only. This maximum possible critical current is difficult to achieve in real junctions. In our opinion, the most reliable way to determine the critical current is to compare the experimental lifetime as a function of the current with the lifetime calculated using numerical simulations [39–41] in the frame of the resistively–capacitively shunted junction (RCSJ) model with additive noise [29]. It is important to use the RCSJ model in the temperature range in which it is valid, that is, above the crossover temperature in the quantum regime.

The lifetime (dark count time) measurements are organized as follows. The current through the junction is quasi-adiabatically ramped up to a given value. After reaching the required bias current, the countdown of lifetimes begins until the moment of jumping to the resistive branch. This cycle is repeated 100–200 times to collect statistics, after which the average value of the switching time and its standard deviation are calculated.

Since the considered Josephson junction is standard and there is no phase-diffusion regime observed (see Figure 3), there is no mixed mode of operation, in which a part of the time there are short voltage pulses due to escapes to the adjacent potential minima and a part of the time the voltage is zero. This makes it easier to determine the lifetime in the numerical model. In this case, the JJ is considered to be switched if the phase exceeds a certain threshold value, usually chosen to the right of the position of the nearest maximum of the potential for a given bias current.

There is a need to use numerical simulations since, in the experiment, we are limited by the time constant of the filters that provide suppression of external interferences. As a result, we cannot measure switching times faster than the time constant, which in our case is about 1 ms. To obtain shorter times, we numerically solve the Langevin equation with noise source [39–40] in the frame of the RCSJ model, which has been proven for classical JJs in the thermal regime [29]. Its applicability is also confirmed for our case by a good overlap with the experimental data.

**Figure 4:**
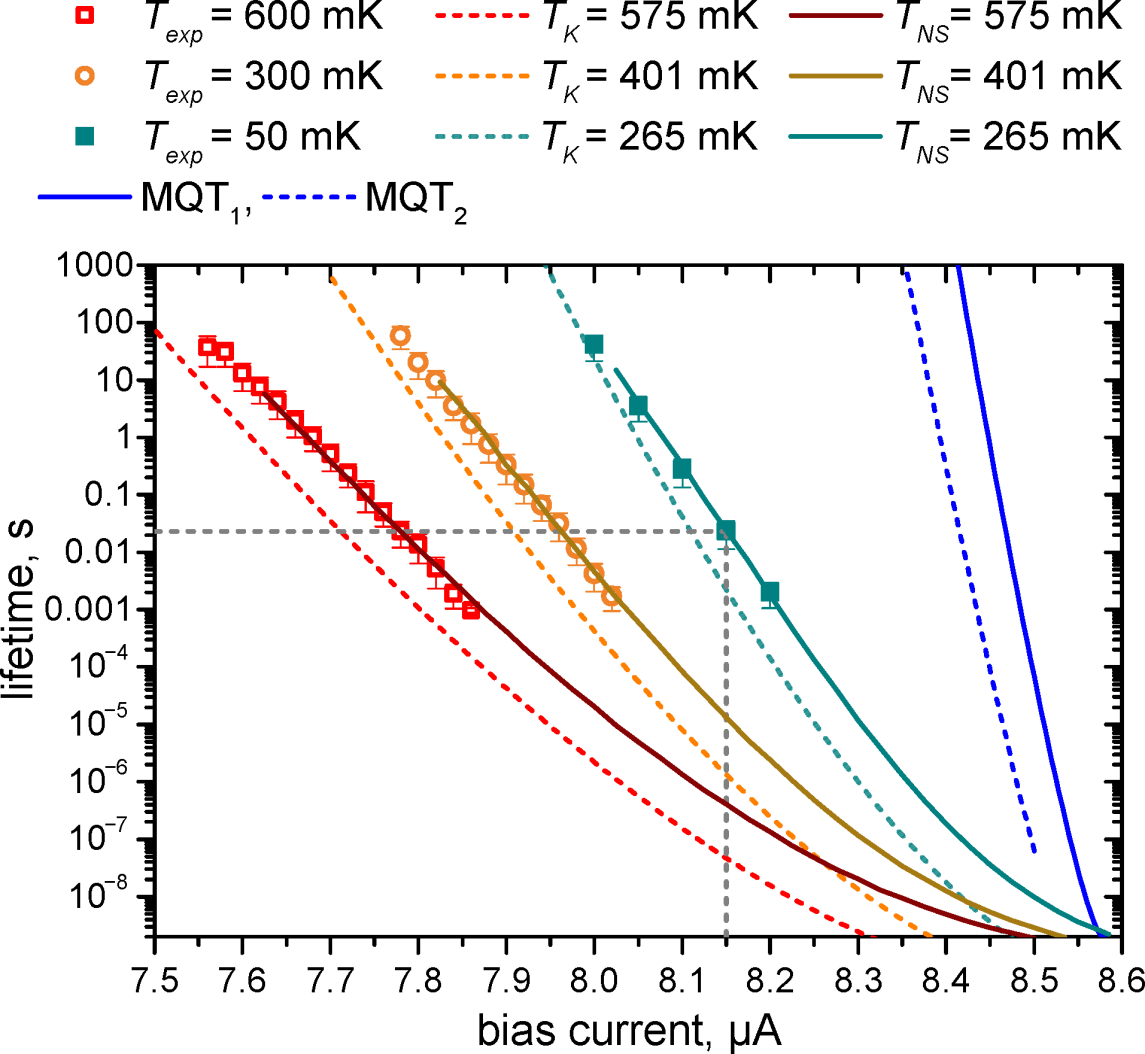
The lifetime of the junction as a function of the bias current at temperatures of 50 mK (green), 300 mK (orange), 600 mK (red). Here, fitting is performed using the approximate Kramers’ formula in Equation 1 (dashed curve) and using the numerical solution of the Langevin equation with noise (solid curve). In the latter case, the agreement is rather good.

It is seen from Figure 4 that the experimental points at 300 and 600 mK agree well with the simulation results if the parameters for numerical calculations are 401 mK, *I*_c_ = 8.536 μA and 575 mK, *I*_c_ = 8.51 μA, respectively. The normal resistance and the capacitance are *R*_N_ = 29 Ω and *C* = 2700 fF.

It is interesting to note that even the curve for 50 mK is well-fitted if the critical current is set to 8.586 μA and the temperature is 265 mK, which is close to the crossover temperature deduced from Figure 3. For the same parameters, the lifetime was calculated with the well-known Kramers’ formula [42–47], modified for intermediate damping values [48–49]:


[1] $$\tau =\frac{a_{t}\exp\left(\Delta u/\gamma \right)}{{\left(1-i^{2}\right)}^{1/4}},\;a_{t}=4{\left(\sqrt{1+\frac{\alpha \gamma }{3.6\sqrt{1-i^{2}}}}+1\right)}^{-2}$$

The used notations are the following: *i* = *I*_bias_/*I*_c_ is the dimensionless bias current with the bias current *I*_bias_ and the critical current *I*_c_, 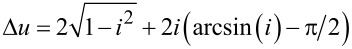 is the potential barrier height, γ = *I*_T_/*I*_c_ is the noise intensity, and *I*_T_ = 2*ekT*/ℏ is the fluctuational current which can be calculated as: *I*_T_ [μA] = 0.042 T [K] [29] for a given temperature *T*. Note that, the thermal current is 2.1 nA for 50 mK and 21 nA for 500 mK, respectively.

The investigated junction also demonstrates a typical Kramers’ dependence of the lifetime, see Figure 4, but the analytical estimates from Equation 1 give an underestimated lifetime compared to a more accurate numerical calculation.

Thus, the critical current at a temperature of 50 mK was determined as 8.586 μA. For this *I*_c_ value, the tunneling time as a function of the bias current was calculated, which is believed to be the reason that, below the crossover temperature, the lifetime stops changing. The results are shown as a solid blue curve if the tunneling occurs from the minimum of the potential profile [49], and as a dotted blue curve if it occurs from the zero-energy level [50]. As can be seen, these curves have a steeper slope than the experimental lifetime at 50 mK. This may indicate that we do not reach the true quantum regime, and the lifetime stops changing with decreasing temperature due to either residual low-frequency interference or overheating. Additional experiments are planned to determine this issue.

The absorption of a photon increases the energy of a JJ by a certain value and may result in switching into the resistive state. Several frequency ranges of effective detection may exist [39] due to resonant activation, and the most efficient switching occurs at signal frequencies of 0.6 from ω_p_ = (2*eI*_c_/ℏ*C*)^1^*^/^*^2^ [40], which is fully consistent with the parameters of the considered experiment. In the current work, we measure the probability of switching initiated by a 10 GHz signal with a fixed duration *t*_sw_ = 0.05 s. The plasma frequency of the junction is 15.6 GHz, while at the bias current of 815 μA, where we presumably see three-photon sensitivity, the resonant frequency ω_r_ of the Josephson junction oscillation circuit ω_r_ = ω_p_(1 − *i*^2^)^1^*^/^*^4^ is 8.8 GHz.

**Figure 5:**
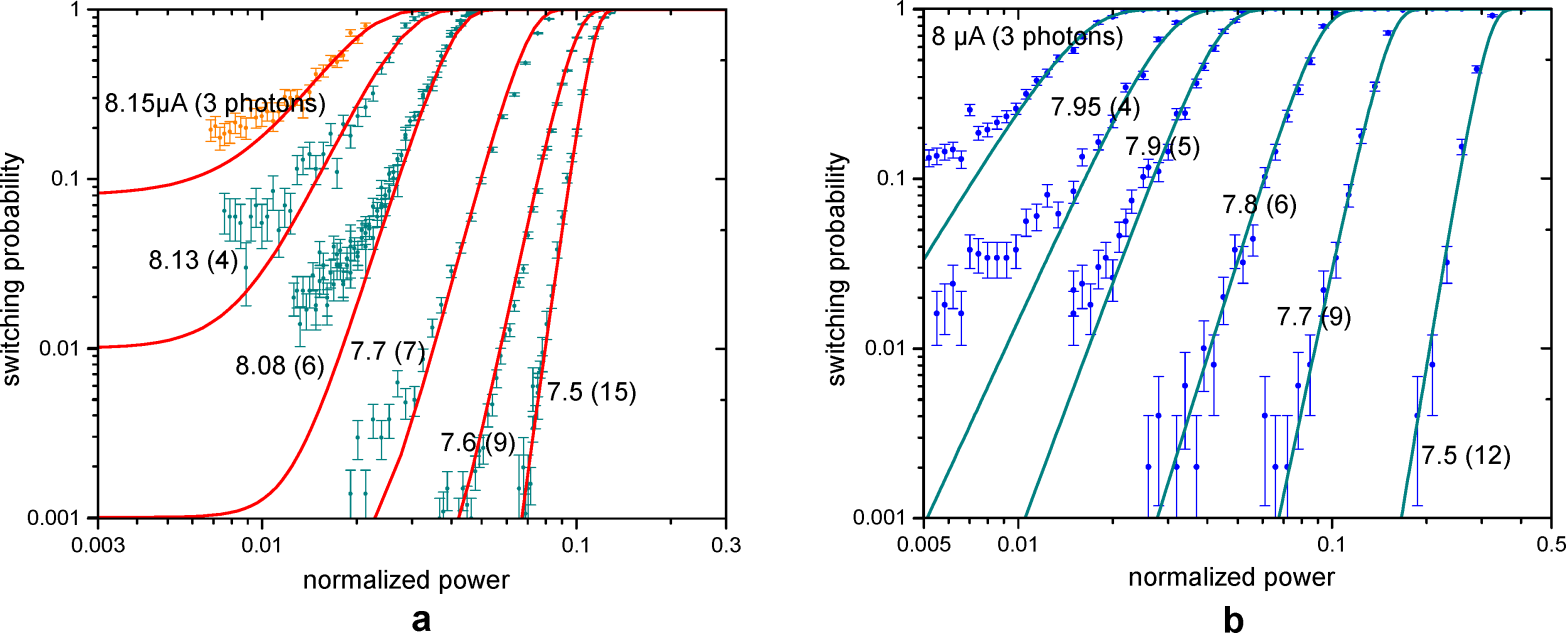
The switching probability of the JJ as a function of the power of the signal (with duration 50 ms) for different bias currents. The dots with error bars are experimental data. For each switching event, the system was first prepared in the initial state by quasi-adiabatically ramping the bias current over 50 ms. If the microwave signal caused a switching to the finite-voltage state during the driving pulse, such event was counted as 1, and 0 otherwise. (a) *T* = 50 mK. The orange dots represent a bias of 8.15 μA. The red fit curves are obtained with the formula in Equation 2. (b) *T* = 500 mK. The green fit curves are obtained with the formula in Equation 2.

The statistics of switching as function of the absorbed power is illustrated in Figure 5a,b for several bias currents and temperatures of 50 and 500 mK, respectively. Each curve in Figure 5 has been collected with (200 to 10^4^) averages of switching events.

The experimental results in Figure 5a,b can be reproduced using the Poisson distribution of photons and the fitting procedure from [23]. To take into account that there may be several switching attempts during the pulse, for the fitting procedure, the signal with duration *t*_sw_ is divided into *M* shorter pulses δ*t*, each of which contains only one group of photons, and we can assume only one switching attempt. The average number of photons *N* in each group is determined by the Poisson distribution. The switching probability in δ*t* is the sum of the products of the Poisson distributions that photons are contained in the signal and the switching probabilities *q*[*i*] due to absorption of *i* photons. The switching probability *p*_sw_ is the result of *M* switching attempts over time δ*t*:


[2]

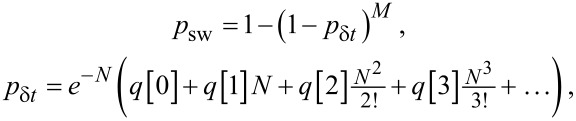



where *q*[0] is the probability of the erroneous detection without a photon, and *q*[1], *q*[2], *q*[3], … are the detection efficiencies for one, two, three, … photons, respectively.

The slope of the fitting curves is set by the number of photons, triggering the switching. The position on the power axis is determined by the effective system response time δ*t* and by the efficiency of switching *q*. The fitting curves in Figure 5a are obtained with δ*t* = 0.3 ns for slope 3 and δ*t* = 5.7 ns for slope 15.

The curve with slope 3 fits the experimental data for the bias current of 8.15 μA quite well if the *q*-array is [5 × 10^−10^, 5 × 10^−10^, 5 × 10^−10^, 0.002, 0.13, 1, 1, …]. Therefore, the probability of switching due to the absorption of 3 photons is 0.002. In Figure 2b the barrier height is compared with the energy of one photon. The potential profile is calculated for the critical current of 8.586 μA. Photon frequency and energy are, respectively, 10 GHz and 6.8 × 10^−24^ J. The energies of three and even five photons are smaller than the barrier height. However, switching may still occur due to either resonant tunneling or resonant activation effects [22,39–40,49,51].

With the critical current of 8.586 μA, the barrier height for bias currents in the range of 7.5–8.08 μA equals to the energy of 35 to 11 photons. This number is quite close to the number we get from the fit of probability versus power slopes, that is, 15 to 3. Even if the total energy of absorbed photons is less than the barrier height, the probability to switch to the resistive state by tunneling increases significantly.

In Figure 5, one can see how the switching probability evolves with increasing temperature from 50 to 500 mK. The difference is not very large because at 50 mK the effective temperature was rather 265 mK, according to numerical simulations, and the thermal current at 500 mK is much smaller than the critical current. There is still three-photon sensitivity with efficiency 0.01, but for a slightly lower bias current of 8 μA. Curves for other bias currents can be fitted with slopes 4, 5, 6, 9, and 12.

The small difference between the results at 50 mK (265 mK) and 500 mK can be understood from Figure 2b. The superconducting gap decreases by a few percent due to the temperature increase from 265 to 500 mK according to the BCS model. It leads to a minor decrease in the JJ critical current. Thus, the qualitative picture remains the same for 265 and 500 mK: The height of the potential barrier is still several times larger than the thermal energy and the energy of a single 10 GHz photon.

## Conclusion

We have presented an experimental study of a Josephson junction with an area of 60 μm^2^ and a critical current of 8.6 μA for application as a single photon counter in the microwave frequency range. Using a strongly attenuated 10 GHz harmonic signal with Poisson distribution of photons as the photon source, three-photon sensitivity with an efficiency of 0.002 and a dark count time of 0.02 s has been shown.

From the analysis of the lifetime, we see that there is a room for improvement of the sensitivity if residual low-frequency noise or overheating of the junction could be decreased. The source of the issue and the way of its suppression need to be investigated in further experiments.

Comparing the obtained results for the considered sample with other small-area junctions [7,23], we can conclude that the optimal critical current range, allowing for improvement of both sensitivity and dark count time, is about hundreds of nanoamperes as predicted in [6]. Such junctions are currently being measured.
